# Food Additives, Gut Microbiota, and Irritable Bowel Syndrome: A Hidden Track

**DOI:** 10.3390/ijerph17238816

**Published:** 2020-11-27

**Authors:** Emanuele Rinninella, Marco Cintoni, Pauline Raoul, Antonio Gasbarrini, Maria Cristina Mele

**Affiliations:** 1 UOC di Nutrizione Clinica, Dipartimento di Scienze Mediche e Chirurgiche, Fondazione Policlinico Universitario A. Gemelli IRCCS, Largo A. Gemelli 8, 00168 Rome, Italy; 2 Scuola di Specializzazione in Scienza dell’Alimentazione, Università di Roma Tor Vergata, Via Montpellier 1, 00133 Rome, Italy; marco.cintoni@gmail.com; 3 UOSD di Nutrizione Avanzata in Oncologia, Dipartimento di Scienze Mediche e Chirurgiche, Fondazione Policlinico Universitario A. Gemelli IRCCS, Largo A. Gemelli 8, 00168 Rome, Italy; pauline.raoul1@gmail.com (P.R.); mariacristina.mele@unicatt.it (M.C.M.); 4 UOC di Medicina Interna e Gastroenterologia, Dipartimento di Scienze Mediche e Chirurgiche, Fondazione Policlinico Universitario A. Gemelli IRCCS, Largo A. Gemelli 8, 00168 Rome, Italy; antonio.gasbarrini@unicatt.it; 5 Dipartimento di Medicina e Chirurgia Traslazionale, Università Cattolica Del Sacro Cuore, Largo F. Vito 1, 00168 Rome, Italy

**Keywords:** food additives, IBS, microbiota, artificial sweeteners, polyols, emulsifiers, carboxymethylcellulose, polysorbate-80, titanium dioxide, food preservatives, personalized medicine

## Abstract

The interactions between diet, gut microbiota, and irritable bowel syndrome (IBS) have many complex mechanisms that are not fully understood. Food additives are one component of the modern human diet that deserves attention from science and government policies. This review aims at identifying the current knowledge about the impact of food additives on gut microbiota and their potential role in the development of IBS. To date, few data on the effect of food additives on gut microbiota in IBS patients are available. However, exposure to food additives could induce the dysbiosis and dysregulation of gut homeostasis with an alteration of the gut barrier and activation of the immune response. These microbial changes could exacerbate the gut symptoms associated with IBS, such as visceral pain, low-grade inflammation, and changes in bowel habits. Some additives (polyols) are excluded in the low fermentable oligo-, di- and monosaccharide, and polyol (FODMAP), diets for IBS patients. Even if most studies have been performed in animals, and human studies are required, many artificial sweeteners, emulsifiers, and food colorants could represent a potential hidden driver of IBS, through gut microbiota alterations. Consequently, food additives should be preventively avoided in the diet as well as dietary supplements for patients with IBS.

## 1. Introduction

The prevalence of irritable bowel syndrome (IBS) in Western countries ranges from 10% to 15%, while lower prevalence rates have been reported in non-Western and developing countries [1]. To date, a specific biomarker for identifying IBS remains unknown, and its definition is entirely based on symptoms [2,3]. Patients with IBS typically experience abdominal discomfort or pain related to defecation with stool changes including diarrhea, constipation, or alternating constipation and diarrhea. Although the pathophysiology of IBS remains poorly understood, it has been suggested that genetic susceptibility, visceral hypersensitivity, food intolerance, altered gut–brain axis, gut dysmotility, immune dysfunction, and dysbiosis are the hallmark characteristics of IBS [4]. Diet is one of the key environmental factors that could induce gut dysbiosis directly influencing host homeostasis and immunological processes [5]. In this context, the interplay between diet and microbiota might have an essential role in IBS etiology [6].

Since the last few decades, the Westernization of eating habits has led to the increasing consumption of food additives being incorporated in almost all processed food [7]. Among them, salt is one of the most important natural additives used for food preservation. High salt consumption is well-known to alter gut microbiota composition and fecal short-chain fatty acids (SCFA) production [8], impacting the gut–immune axis through the modulation of T helper 17 cells [9] and promoting local and systemic tissue inflammation, which may lead to hypertension [10] and obesity [11]. On the other side, there are many other artificial substances added intentionally to food products to improve appearance, preserve flavor, and extend shelf life. The use of additives in food processing is regulated by the Food and Drug Administration (FDA) in the United States and the European Food Safety Authority (EFSA) in Europe. However, the outdated evaluation of the effect of food additives on human health is raising concerns. Currently, the EFSA is re-evaluating the safety of all food additives authorized for use before 20 January 2009, and final results should be obtained at the end of this year [12].

In this context, the effects of artificial food additives on microbiota have recently received much attention [13]. Indeed, emerging evidence suggests interactions between artificial food additives and microbiota, which may affect host health [14,15,16]. Furthermore, many preclinical studies have recently associated the increased and prolonged consumption of food additives with the development of colitis [17] and metabolic diseases [18,19], which is closely associated with detrimental effects on gut homeostasis.

This review aims to report and discuss the current knowledge about the impact of food additives on gut microbiota and their potential role in the development of IBS. We focused on some food additives, such as non-caloric sweeteners, polyols, emulsifiers, food colorants and preservatives.

## 2. Methods

A systematic literature search was performed across PubMed, Web of Science and Scopus databases from inception to September 2020. The relevant articles were identified and hand-searching was done to check the reference lists and find original as well as additional references. The search terms included “irritable bowel syndrome,” “IBS”, “microbiota”, “microbiome”, “artificial food additives”, “artificial sweeteners”, “polyols”, “acesulfame potassium”, “aspartame”, “saccharin”, “sucralose”, “cyclamate”, “neotame”, “emulsifiers”, “carboxymethyl cellulose”, “polysorbate 80”, “food coloring agents”, “food preservatives”, “benzoic acid”, “sodium benzoate”, “titanium dioxide”, and “sodium nitrite”.

## 3. IBS, a Gut Microbiota-Related Disorder

The onset of IBS-related symptoms often occurs during adolescence and affects more females than males [20]. The “Rome IV Criteria” is a diagnostic approach of IBS requiring that the patient has abdominal pain on average at least 1 day/week, and that pain is associated with two or more of the following characteristics: defecation, change in the frequency of stool, and change in the form (appearance) of the stool [21]. These criteria should be fulfilled for the previous 3 months, with symptoms onset at least 6 months prior to diagnosis [22]. Patients with IBS can be categorized into four major subtypes depending on the predominant stool pattern, including IBS with constipation (IBS-C), IBS with diarrhea (IBS-D), IBS with mixed stool types (IBS-M), and unclassified IBS [21]. Although the pathogenesis is still poorly understood, the underlying mechanisms that could lead to IBS include genetic and epigenetic influence, central nervous system alterations, and dietary and gastrointestinal influences such as gut microbiota alterations, gut motility changes, and low-grade mucosal inflammation and immune activation [23].

Many recent studies have suggested that gut microbiota are a key player in IBS pathogenesis [24,25,26,27]. The gut microbiota constitute a complex changing ecosystem that contains more than thousands of bacterial species and milliards of microorganisms mainly found in the distal small bowel and colon [28]. The anatomical gut barrier includes commensal gut microbiota, the mucus layer, and the intestinal epithelial monolayer. Abnormally increased permeability and alterations of the epithelial barrier and tight junctions were observed in some IBS patients [29]. Commensal bacteria are also key regulators of immune responses. Indeed, gut microbiota may regulate the production of T helper cells, resulting in inflammatory responses through the production of interleukins (IL) [30]. Specifically, *Bifidobacterium infantis* and *Faecalibacterium Praustnizii* could stimulate regulatory T cells (T reg) and the production of the anti-inflammatory cytokine IL-10. Interestingly, higher T cell levels isolated from blood and colonic biopsies were activated in IBS patients compared with healthy controls [31]. Furthermore, numerous studies, summarized in a recent systematic review [32], showed that low-grade mucosal inflammation could be associated with IBS. Moreover, increased levels of pro-inflammatory cytokines and higher numbers of mast cells are located close to enteric nerves in the gastrointestinal mucosa of IBS patients [33]. This condition could be associated with an activation of the immune system and an increased release of pro-inflammatory cytokines from isolated peripheral blood mononuclear cells, specifically in IBS-D patients [34].

Multiple studies investigated the associations between IBS and microbiota composition. Recently, a systematic review of studies compared the α-diversity and gut microbiota variations between patients with IBS and healthy controls [35]. For most studies, the fecal samples of patients with IBS had a lower α-diversity than healthy controls [36,37]. Patients with IBS had an increased Firmicutes:Bacteroidetes ratio, an increased abundance of *Clostridia* and *Clostridiales*, and a decreased abundance of *Bacteroidia* and *Bacteroidales* [36,37,38]. Given these findings, we can hypothesize that these increases or decreases of specific microbial groups could lead to microbial dysbiosis, a potential hallmark of IBS. Furthermore, various studies [29,39] have reported an increased intestinal permeability in patients with diarrhea-predominant IBS (IBS-D).

As such, the gut microbiota are involved in the development of normal gut barrier integrity and gut immune system, but can also play a central role in the dysregulation of gut functions involved in IBS pathophysiology [40].

## 4. Artificial Sweeteners, Gut Microbiota, and IBS

Non-caloric artificial sweeteners (NAS) are synthetic non-nutritive sweeteners characterized by a higher sweetening intensity without increasing caloric intake [41]. NAS are mainly found in soft drinks, snack foods, sugar-free candies, and dairy products. The EFSA has approved various NAS such as acesulfame potassium (K) (E-950), aspartame (E-951), cyclamate (E-952), saccharin (E-954), sucralose (E-955) and neotame (E-961) [42].

### 4.1. Artificial Sweeteners and Gut Microbiota Composition

In rats, saccharin, sucralose and aspartame consumption were shown to be associated with an increase in *Bacteroides spp* and Clostridiales [18]. Palmnas et al. showed that low-dose aspartame consumption (5–7 mg/kg/day in drinking water for 8 weeks) in rats increased total bacteria, including *Clostridium Leptum* and Enterobacteriaceae [43]. Uebanso et al. found equivalent amounts of total bacteria, Firmicutes, Bacteroidetes, Bacteroides, *Clostridium IV* and *Clostridium XIVa* in male C57Bl/6J mice exposed to acesulfame K (15 mg/kg/day in drinking water for 8 weeks) and control groups [44]. Chi et al. [45] reported in male CD-1 mice exposed to neotame (0.75 mg/kg body weight/day for 4 weeks) a significant increase in Bacteroidetes and a significant decrease in Firmicutes compared with controls. The relative abundances of *Ruminococcaceae*, *Lachnospiraceae* such as *Ruminococcus*, *Oscillospira*, *Dorea* and *Blautia* were significantly lower in neotame-fed mice than in controls [45].

In humans, in a recent cross-sectional clinical study of 31 participants, an analysis of the fecal samples of adults consuming aspartame and acesulfame K did not report increased bacterial abundance, but decreased bacterial diversity, compared with those of non-consumers [46]. In seven participants consuming 1.7–33.2 mg/day of acesulfame K for 4 days, the median Bacteroidetes:Firmicutes ratio did not change, but bacterial diversity statistically differed compared with non-consumers [46].

According to a recent systematic review of human and animal studies [47], the consumption of artificial sweeteners, including acesulfame K, aspartame, saccharin, sucralose, cyclamate, and neotame, is associated with gut dysbiosis [47]. Further studies and large randomized controlled trials are needed to investigate whether the intestinal microbiota variations reported in animals are present in humans.

### 4.2. Effects of Artificial Sweeteners on Gut Functions and Metabolism

Various studies explored the functional effect of NAS on the gut microbiota and host. They demonstrated an association of NAS consumption with increased insulin resistance and glucose intolerance [18,43,48]. Interestingly, Suez et al. showed in saccharin-consuming mice a significant increase in glycan degradation compared with control mice [18]. Given that gut bacteria fermented glycans into SCFAs [49], saccharin exposure could decrease SCFAs production. Recently, Farup et al. found that a NAS-enriched diet could be associated with a reduction in butyrate production in morbidly obese adults [50]. Butyrate has key anti-obesity effects induced by reducing appetite and activating brown adipose tissue via the gut–brain neural circuit [51]. As such, these results highlight the potential adverse effects of some NAS on the fermentation of glycans and the SCFAs’ production, leading to metabolic disorders. Furthermore, Bian et al. [48] analyzed mouse livers after 6 months of saccharin administration, reporting significant liver inflammation with an elevated gene expression of TNF-α in the liver of saccharin-treated mice compared with control. TNF-α is a key cytokine in inflammation, mainly produced by macrophages induced by pathogen-associated molecular patterns (PAMPs), such as lipopolysaccharides (LPS). Moreover, the expression of TNF-α can activate NFκB pathways and induce cell damage and inflammatory responses.

### 4.3. Impact of NAS Induced Microbiota Changes in IBS

To date, to our knowledge, a direct link between NAS-induced microbiota changes and IBS has not been closely studied. However, some studies [52,53,54] have demonstrated a significant increase in Bacteroides in IBS-D patients. A recent systematic review of 24 clinical trials [55] showed microbiota variations associated with IBS, with an increase in *Enterobacteriaceae*, *Lactobacillaceae* and *Bacteroides*. As previously described, NAS consumption could increase *Bacteroides* [18] and *Enterobacteriaceae* [43] in rodent models. *Enterobacteriaceae,* such as *Escherichia coli*, *Klebsiella spp.* and *Proteus spp.*, belong to the Proteobacteria phylum and are localized close to the mucosal epithelium. An infection by a pathogen [56], chemically induced colitis [57] or deficiencies in host immunity [58] could cause gut inflammation and could stimulate the growth of *Enterobacteriaceae*. Consequently, even if it is still difficult to identify specific intestinal bacteria associated with NAS consumption and IBS, it appears that NAS could be associated with dysbiosis, gut inflammation, and consequently symptoms associated with IBS subtypes.

## 5. Polyols, Gut Microbiota and IBS

Polyols are sugars. They are found naturally in some fruits, vegetables, and mushrooms. The FDA and EFSA approved different polyols such as erythritol (E-968), maltitol (E-965), sorbitol (E-420), and xylitol (E-967) for use as sweeteners. They are mainly used in chewing gum, candies, and beverages.

### 5.1. Polyols and Gut Microbiota

Xylitol (at the dosage of 40 mg/kg body weight/day) could decrease the abundance of fecal Bacteroidetes and *Barnesiella,* and stimulate the growth of Firmicutes and *Prevotella* in mice fed a high-fat diet [59]. As regards maltitol, a study of 40 human volunteers consuming low-energy chocolate containing 34.2 g of maltitol plus polydextrose found a significant increase in the abundance of fecal *Bifidobacteria*, *Lactobacilli* and SCFAs levels [60]. However, to date, the specific effects of maltitol on gut microbiota remain almost unexplored. Additionally, erythritol, a polyol completely non-fermentable by the human gut microbiota, is to date considered a safe additive [41,61]. Indeed, erythritol is absorbed through the small intestine with very low metabolism, and 90% of the substance is excreted unchanged in the urine [62]. Thus, a limited amount of erythritol reaches the colon and could potentially affect the gut microbiota [63]. As regards lactitol, an in vitro study showed—at the dosage of 2 mg/L—that it could reduce *Enterobacteriaceae* abundance in feline fecal cultures [64]. In humans, fecal samples of 36 healthy adults consuming 2 × 10 g/d of lactitol or lactulose over 9 weeks were analyzed. Lactitol decreased bacterial populations of *Bacteroides, Clostridium* and *Eubacterium* compared with the lactulose group [65]. Another study by Finney et al. [66] analyzed fecal samples from 75 healthy adults before and after 7 days of consumption of low doses of lactitol (25 g tablets/day of milk chocolate containing 10 g sucrose:lactitol in ratios of 10:0, 5:5 or 0:10), showing that lactitol could increase the abundance of *Bifidobacteria*. Furthermore, a recent study showed that lactitol consumption in mice could enhance the growth of *Akkermansia* [67]. *Akkermansia* is a bacteria belonging to the Verrucomicrobia phylum, which improves the inflammatory response and reduces insulin resistance in obese and diabetic patients [68], protects the intestinal epithelial cells, and preserves the mucosal barrier function [69]. Thus, all these findings suggest that lactitol consumption could positively influence the gut microbiota’s composition. Regarding sorbitol, a rat model study compared the effects of consumption of water plus sorbitol 10% with those of consumption of water plus fructooligosaccharides (FOS) 10% for 16 days [70]. The authors showed that both sorbitol and FOS exposures increased *Lactobacillus reuteri* cell numbers. Interestingly, sorbitol intake also maintained the levels of *Lactobacillus* sp. *AD102,* and significantly increased butyrate levels [70]. As such, in animal models, sorbitol could modify gut microbiota activity and contribute to gut homeostasis. However, further studies are needed to define the specific effects of sorbitol on the gut microbiota in humans.

### 5.2. Impact of Polyols Induced Microbiota Changes in IBS

No studies assessing the effects of polyols on the gut microbiota of patients with IBS were found. However, data are available on the effects of diet on the microbiota of IBS patients. Polyols are included in the category of the so-called “FODMAP” (fermentable oligo-, di- and monosaccharides, and polyols) diet. The term “FODMAP” was created by the Monash University in 2004 to define highly fermentable carbohydrates and polyols whose restriction would improve the outcome in IBS treatment [71]. In recent years, the FODMAP diet has become a valuable treatment option for IBS patients [72]. Halmos et al. studied the effects of FODMAP diet on the microbiota of 30 IBS patients reporting unchanged SCFA levels and a reduction of 47% of the total bacterial abundance in IBS patients following a low-FODMAP diet compared with those following a habitual diet [73]. Several other studies showed bacterial variations in patients following low-FODMAP diets, such as decreases in *Akkermansia muciniphila* [73], *Clostridium cluster IV* [73], *Propionibacteriaceae* [74], *Ruminococcus gnavus* [74] and *Bifidobacteria* [73,75,76] compared with patients following the control diet. Larger studies are needed to further understand the long-term effects of low-FODMAP diets, and especially the effects of polyols, such as sorbitol, on intestinal microbiota composition and IBS.

## 6. Emulsifiers, Gut Microbiota and IBS

Emulsifiers are amphiphilic molecules used either to facilitate processing or to improve the texture and shelf life of processed foods [77]. They are found in various processed foods such as sauces, puddings, margarine, and ice-creams. They are highly prevalent in the Western diet.

### 6.1. Effect of Emulsifiers on Microbiota Composition

Various studies investigated emulsifiers such as carboxymethylcellulose (CMC) and polysorbate-80 (P80). An animal study investigated the variations of gut microbiota in IL-10 gene-deficient mice fed with 2% CMC solution for 3 weeks [78]. CMC-treated IL-10 gene-deficient mice reported an increase in total bacteria abundance in the ileum in treated mice compared with control mice. Specifically, a decrease in *Eubacterium rectale* in the ileum and jejunum and an increase in Bacteroides were found in mice treated with CMC. Chassaing et al. examined the direct impact of CMC on the microbiota using the mucosal simulator of the human intestinal microbial ecosystem (M-SHIME) model [79]. After CMC treatment for 13 weeks, the abundances of Proteobacteria and Firmicutes decreased significantly, and the abundance of Bacteroidetes increased. Similarly, the impact of P80 on gut microbiota composition was investigated. An animal study [17] showed that P80 intake had no significant effect on the total fecal bacteria in mice. However, a recent study [80] in mice treated with P80 (1.0% drinking water solution for 8 weeks) demonstrated that P80 increased the Gamma-proteobacteria abundance, decreased the α-diversity in the small intestine, and did not impact α-diversity in the colon [80]. Viennois et al. [15] also showed that the relative abundances of Proteobacteria and Firmicutes in P80-treated mice decreased significantly, and the abundance of Bacteroidetes increased compared with controls. Glycerol monolaurate is another emulsifier recently investigated in C57BL/6 mice [81]. Glycerol monolaurate significantly changed the β-diversity with a decrease in *Akkermansia muciniphila* and *Lupinus luteus*, and an increase in *Bacteroides acidifaciens* and *Escherichia coli* [81]. Recently, Elmen et al. [82] studied the in vitro effects of glycerol monoacetate, glycerol monostearate, glycerol monolaurate, propylene glycol monostearate, and sodium stearoyl lactylate on fecal microbiota. Sodium stearoyl lactylate exposure (0.025% water drinking solution) reduced the abundances of *Clostridiaceae*, *Lachnospiraceae* and *Ruminococaceae*, and increased the abundances of *Bacteroidaceae* and *Enterobacteriaceae* [82]. To sum up, emulsifiers could directly alter the microbiota composition in mice/rats and in in vitro studies, but how these findings translate to humans needs further investigation.

### 6.2. Emulsifiers and Gut Functions

Chassaing et al. [17] showed the huge impact of P80 and CMC on fecal bacterial composition with an increase in *Ruminicoccus gnavus* and *Akkermansia muciniphila*; these changes have been associated with increased intestinal permeability, LPS levels, and flagellin circulating levels in mice as a result of a reduction in the intestinal mucus thickness. LPS represents the primary bacterial component encountered by the host immune system, while flagellin recently emerged as a potent immune activator, shaping both the innate and adaptive arms of immunity during microbial infections [83]. In germ-free mice receiving fecal transplantation from P80- and CMC-treated animals, a low-grade intestinal inflammation was observed [17]. Viennois et al. highlighted the role of these emulsifiers in intestinal inflammation-promoting colon carcinogenesis in mice [15]. Specifically, in mice exposed to P80 for 4 weeks, another study [84] reported the lower expression of mucin-2 with an increase of lipocalin-2 (LCN2), LPS, and flagellin, associated with a higher tumor incidence. An in vitro study confirmed these findings, showing an increase in the expression of the flagellin gene and LPS concentration in mice using the M-SHIME model and microbiota transplantation [79]. Furthermore, a recent rodent study [80] showed that CMC as well as P80 aggravated ileitis and increased the number of sulfide-producing bacteria. The high concentration of sulfide produced by sulfide-producing bacteria such as *Bilophila wadsworthia* may reduce disulfide bonds in the mucus, lysing the network of the polymeric protein MUC2 (oligomeric mucus gel-forming) [85]. MUC2 is secreted by goblet cells and has a key role in mucus layer stability and mucosal repair [86].

### 6.3. Impact of Emulsifiers in IBS

Mucosal barrier alterations could allow the passage of an increased abundance of luminal antigens which, in turn, activates the mucosal immune responses involved in diarrhea symptoms. As previously described above, emulsifiers could impact microbiota composition, including an increase in the sulfate-reducing bacteria that could exacerbate the hallmark symptoms of IBS. Appropriately, sulfate-reducing bacteria reduce sulfate to hydrogen sulfide (H_2_S), which was recently recognized as a gaseous neuromodulator/neurotransmitter that modulates intestinal inflammation [87]. We can hypothesize that sulfate-reducing bacteria play a significant role in modulating visceral pain in IBS by producing H_2_S. As such, the consumption of emulsifiers by IBS patients could increase gut barrier permeability and intestinal inflammation, with an increase in LPS and flagellin and a decrease in MUC2. Holder et al. [88] confirmed that CMC and P80 exposure (drinking water containing CMC or P80 (1%) for 12 weeks) could induce chronic intestinal inflammation, and alter gut microbiota composition in mice. Further work is needed to better understand the interplay between emulsifiers, gut microbiota, and epithelial mucosa in the pathogenesis of IBS.

## 7. Food Colorants, Gut Microbiota, and IBS

### 7.1. Food Colorants and Gut Microbiota Composition

Food colorants are mainly added to cheeses, sauces, skimmed milk, ice-creams, pastries, sweets, chocolates, and chewing-gum [89]. Titanium dioxide (TiO_2_) is commonly used as a whitening or brightening agent in food products. TiO_2_ is referred to as E171 in Europe. In recent years, various studies have attempted to identify the impact of the oral consumption of TiO_2_ on gut microbiota composition in mice and humans. In mice treated with TiO_2_, a significant increase in Firmicutes [90] and a decrease in Bacteroidetes [91] were observed compared with controls. In particular, the abundance of *Barnesiella*, an anaerobic bacterium belonging to the Porphyromonadaceae family of the Bacteroidetes phylum, is significantly affected by TiO_2_ exposure (160 mg/kg/day for 28 days) [91]. *Barnesiella* could be a key protective intestinal bacterium, removing harmful bacteria from the intestine [92]. Mu et al. [93] found a significant decrease in *Lactobacillus* and *Bifidobacterium* in adult C57BL/6J female mice fed with a diet containing 0.1% TiO_2_ for 3 months. Overall, animal studies found little effect of TiO_2_ on total microbial diversity. In vitro studies confirm this trend, showing that TiO_2_ might not dramatically reshape the human microbiota in a model of simplified human microbiota [94,95]. However, TiO_2_‘s impact on human microbiota over a long period has not been investigated.

### 7.2. Food Colorants and Gut Functions

Food colorants such as TiO_2_ could impact gut homeostasis. Indeed, recently, various in vitro and animal studies have demonstrated potential associations between TiO_2_ exposure and functional adverse effects on gut microbiota. In rats orally exposed for one week (10mg/kg bw/day), TiO_2_ was detected in the immune cells of the Peyer’s patches and regulatory T cells involved in inflammatory responses [96]. Specifically, after TiO_2_ exposure, the stimulation of immune cells isolated from Peyer’s patches showed a decrease in T helper (Th)-1 cells, an increased IFN-γ secretion and an increased Th1/Th17 inflammatory response [96]. Ortega et al. went further, showing possible associations between E171 exposure and the development of intestinal diseases and colorectal cancer in rodents [97]. As regards possible mucus-related disorders, Talbot et al. showed in vitro that TiO_2_ could be captured by intestinal mucus; however, in vivo, two different TiO_2_ exposures (0.1 mg/kg bw/day for 7 days and 10 mg/kg bw/day for 60 days) did not cause variations in SCFAs levels [98]. Thus, given that SCFAs levels (in particular butyrate) are involved in mucin synthesis and mucus properties [99], in vivo, TiO_2_ exposure might not be associated with mucus barrier impairment. On the other hand, Pinget et al. [100] studied different dosages of TiO_2_ (2, 10, 50 mg/kg body weight/day) in mice and reported a reduction in SCFAs levels upon high-dose exposure, a decrease in mucus-related gene expression (MUC2), an increase in inflammatory response and an alteration of colonic crypt length. All these results highlight the need for further research to focus on the influence of TiO_2_ on microbiota and gut health.

### 7.3. Impact of Food Colorants Induced Microbiota Changes in IBS

As previously described, a recent study compared the effects of micro-TiO_2_ and nano-TiO_2_ exposures on the murine intestinal tract, and found a significant bacterial decrease at both the family and genus level, including the *Verrucomicrobiaceae* family, *Akkermansia* genus, *Porphyromonadaceae* family, and *Barnesiella* genus. Interestingly, with the increase in exposure dose, the abundance of *Barnesiella* showed a significant dose-dependent decrease [91]. In parallel, Chen et al. [101] investigated the effect of a novel prebiotic blend (PB)—composed of fructooligosaccharides (FOS), galactooligosaccharides (GOS), inulin, and anthocyanin—on the development of IBS in mice. PB product in IBS mice could positively impact gut microbiota and modulate the immune response. Among gut microbiota changes, the level of the genus *Barnesiella* significantly decreased in the post-infectious IBS group, while *Barnesiella* increased in the PB pre-treatment group. Moreover, in the development of colitis in IL-10−/− mice, higher levels of *Barnesiella* are associated with lower activity levels of the disease [102]. These observations indicate that the genus *Barnesiella* could protect the intestinal tract from pathogen infections and play a key role in immunomodulation. Thus, all these findings suggest that dysbiosis due to the exposure to food colorants such as TiO_2_ could lead to microbiota dysbiosis, with variations of specific family and genus bacteria potentially involved in the pathogenesis of IBS.

## 8. Preservatives, Gut Microbiota and IBS

Food preservative additives are natural or synthetic substances that delay degradation in foods [103]. They can inhibit the growth of bacteria, fungi, or antioxidants, and inhibit the oxidation of food constituents. We focused on antimicrobial artificial preservatives, including sodium benzoate, sodium nitrate and sodium nitrite.

Although the benefits and safety of artificial preservatives are debated among food scientists and toxicologists, little is known about the effect of food preservatives on the microbiota. Only a recent study [104] found a significant impact of a mixture of sodium benzoate, sodium nitrite, and potassium sorbate consumption on mice colonized with a human microbiome, highlighting an overgrowth of Proteobacteria and a decrease in *Clostridiales*. The same authors further examined the susceptibility of gut microbiota to sodium benzoate, sodium nitrite and potassium sorbate, and their combinations, using a broth microdilution method [105]. They found that gut bacteria with anti-inflammatory properties, such as *Clostridium tyrobutyricum* or *Lactobacillus paracasei*, were significantly decreased compared with intestinal bacteria with proinflammatory or colitogenic properties, such as *Bacteroides taiotaomicron* or *Enterococcus faecalis* [105]. A randomized controlled trial showed that supplementation with *Lactobacillus paracasei CNCM I-1572* for 18 weeks could modulate gut microbiota structure/function and reduce immune activation in IBS patients [106]. Indeed, after treatment, the IL15 levels significantly decreased compared with a placebo, suggesting a key role of *Lactobacillus paracasei* in the restoration of intestinal regulation and mucosal integrity [107].

## 9. Conclusions: Food Additives, a Hidden Driver of IBS

**Figure 1:**
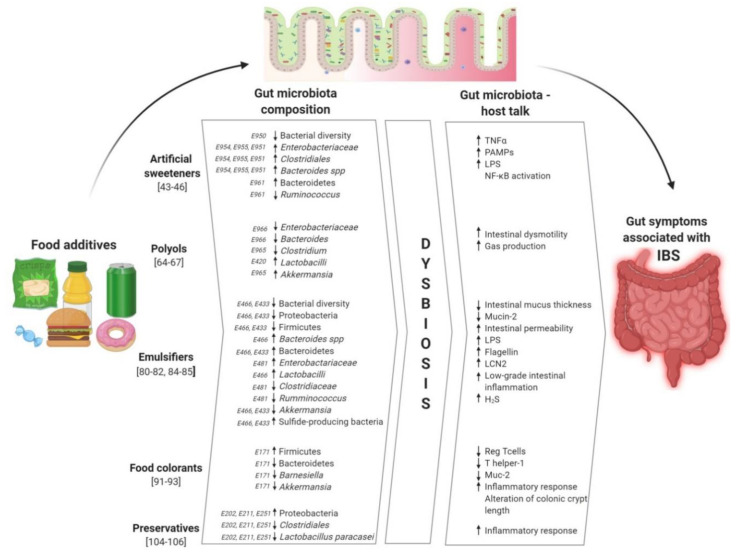
Food additives, gut microbiota, and irritable bowel syndrome (non-exhaustive list of food additives). Abbreviations: E171, titanium dioxide; E202, potassium sorbate; E211, sodium benzoate; E251, sodium nitrate; E420, sorbitol; E433, polysorbate 80; E466, carboxymethyl cellulose; E481, sodium stearoyl-2-lactylate; E950, acesulfame potassium; E951, aspartame; E954, saccharin; E955, sucralose; E961, neotame; E965, maltitol; E966, lactitol; LCN2, lipocalin-2; IBS, irritable bowel syndrome; LPS, lipopolysaccharides; Mucin-2, oligomeric mucus gel-forming protein encoded by the MUC2 gene; NFκB, nuclear factor-kappa B; PAMPs, pathogen associated molecular patterns; Reg T cells, regulatory T cells; TNF, tumor necrosis factor.

All these findings provide valuable insights regarding the potential impact of food additives on the gut microbiota composition potentially involved in the development of IBS (Figure 1). Indeed, exposure to food additives such as emulsifiers or artificial sweeteners alters the composition of gut microbiota in animal studies, even if further studies are needed in humans. Furthermore, polyols are among “FODMAPs”, which are closely associated with IBS symptoms. Gut microbial diversity is a significant indicator of human health, and dysbiosis may be associated with gut dysfunction, leading to host inflammation and numerous non-communicable diseases, as well as IBS. Thus, food additives may substantially impact not only the composition of the microbiota but also its functions. On the other hand, IBS is clearly associated with an altered balance of gut microbiota composition, affecting gut functions.

Given that the consumption of ultra-processed food has increased over the last few decades and food additives are widely used in many such products, we can hypothesize that food additive-induced microbiota alterations might be one of the reasons for the growing incidence of IBS in Western countries. In daily life, IBS patients as consumers are regularly exposed to the food additives present in more than 50% of food products, as reported in a recent analysis of the co-occurrence of food additives on the French market [108]. Moreover, more than 10% of the food products contained five or more food additives. This “cocktail effect” remains overlooked, and further studies should focus on synergies between food additives. Another major issue is the lack of data about the daily intake of these substances. Indeed, although the food safety authorities determined an acceptable daily intake (ADI) for each category of food additives, the ingredient list offers little information for the amounts of additives in food products. Besides, we know that in toxicology, the relationship between the dose of the substance and the response is crucial. In this context, these issues highlight the strong need to understand how a sweetener, emulsifier, or a food preservative precisely impacts a phylum or family of gut bacteria, and to assess any potential synergistic effects among commonly used food additives on gut homeostasis.

As such, to date, although food additives have not been studied specifically in patients with IBS, food additive consumption through the diet and dietary supplementation should be limited. Future studies are required to evaluate the effects of food additives on the human gut microbiota, and to characterize the direct/indirect underlying mechanisms involved in the pathogenesis of IBS.
